# Evidence of Bacterial Co-Infection in Endangered Yangtze Sturgeon (*Acipenser dabryanus*)

**DOI:** 10.3390/biology14111498

**Published:** 2025-10-27

**Authors:** Senyue Liu, Yang Feng, Zhipeng Huang, Chengyan Mou, Qiang Li, Yongqiang Deng

**Affiliations:** 1 Fisheries Research Institute, Sichuan Academy of Agricultural Sciences, Chengdu 611731, China; liusenyue@scsaas.cn (S.L.);; 2 Aquatic Health and Intelligent Aquaculture Key Laboratory of Sichuan Province, Chengdu 611130, China

**Keywords:** *Acipenser dabryanus*, bacterial, co-infection, histology, disease

## Abstract

**Simple Summary:**

In 2024 summer, the endangered Yangtze sturgeon in China suffered large-scale deaths, which was an urgent problem to solve. This study aimed to identify the cause of the disease and provide a basis for protecting the Yangtze sturgeon and preventing such diseases. The findings revealed that the bacterial co-infection (Streptococcus iniae, Klebsiella pneumoniae, Edwardsiella tarda, and Bacillus cereus) triggered by high temperature and high humidity environmental stress was closely related to this large-scale death event. And these bacteria all exhibited resistance to commonly used antibiotics in aquaculture. This was the first time multiple bacterial co-infection was found in Yangtze sturgeon, so protection should shift from focusing on a single bacterium to multiple ones, and ecological prevention measures are needed. These findings help protect this rare species, guide disease prevention in aquaculture, and safeguard aquatic ecological security.

**Abstract:**

The Yangtze sturgeon (*Acipenser dabryanus*) is designated as critically endangered in the IUCN Red List and is a first-class protected species in China. During the summer of 2024, it suffered lethal disease outbreaks. Comprehensive pathological and microbiological analyses were conducted to clarify the etiology. Clinically, infected sturgeon exhibited systemic manifestations including cutaneous ulcers, hemorrhagic septicemia, and diffuse necrosis in liver, kidney and heart tissues. Histopathologically, infected sturgeon showed liver hepatocyte vacuolation/necrosis, renal glomerular atrophy, and cardiac epicardial thickening with lymphocyte/eosinophil infiltration; Gram staining revealed co-localized Gram-positive/negative bacteria in lesions, and TEM identified diverse bacterial morphotypes. Through isolation and molecular identification, four bacterial pathogens were characterized: *Streptococcus iniae*, *Klebsiella pneumoniae*, *Edwardsiella tarda*, and *Bacillus cereus.* Bacterial load detection revealed the presence of these pathogens in lesion tissues. Antimicrobial susceptibility testing indicated multidrug resistance to florfenicol, tetracycline, and ampicillin (commonly used antibiotics in aquaculture), while high sensitivity to ceftazidime, ceftriaxone, and ciprofloxacin was observed. Thus, we infer that sustained high-temperature stress triggered bacterial co-infection is closely related to this large-scale death incident. This is the first evidence of polymicrobial infection in the Yangtze sturgeon, emphasizing the significance of shifting from a single-pathogen perspective to a multi-pathogen framework, and highlighting the urgency of implementing ecological prevention strategies for this species.

## 1. Introduction

Renowned as the “giant panda in water”, the Yangtze sturgeon (*Acipenser dabryanus*) represents a rare and precious freshwater fish unique to China, which has extremely high ecological, economic, and cultural significance [1]. Nevertheless, due to multiple factors such as habitat loss, environmental pollution, overfishing, and the construction of water conservancy projects, the population of Yangtze sturgeon has experienced a drastic decline [2]. The species was officially listed as critically endangered in the International Union for Conservation of Nature (IUCN) Red List in 2010 [3], by the time of this study (summer 2024), 14 years had passed since this endangered classification. To mitigate population decline, targeted conservation measures have been implemented over this period, with artificial breeding, stock enhancement, and release programs emerging as core strategies to safeguard and restore wild populations.

Unfortunately, bacterial diseases remain a major threat in artificial breeding systems, frequently endangering Yangtze sturgeon’s health and survival, and thus limiting effective population recovery [4,5]. However, previous studies on the pathogenicity of the Yangtze sturgeon were very limited, and they mainly focused on the infection of a single type of bacteria (such as *Aeromonas hydrophila* and *Edwardsiella tarda)* [4,5]. This failed to elucidate the mechanism of disease outbreaks characterized by “complex symptoms and high antibiotic resistance’’ in aquaculture, nor did it address the ecological risks of multi-bacterial cross-host transmission.

Bacterial co-infection, defined as the simultaneous infection of a single host by two or more bacterial species (including pathogenic agents and/or opportunistic colonizers), is prevalent in aquaculture [6]. Such co-infections not only increase the complexity of the diseases and the challenges in diagnostics, but also potentiate the development of multidrug resistance (MDR) in bacteria, thereby impeding effective disease control [7]. During the cultivation of Yangtze sturgeon, there exists a wide variety of pathogenic bacteria triggering bacterial diseases, with mixed, cross, secondary, and co-infections being common [5]. However, the etiology of severe outbreaks remains poorly understood.

In the summer of 2024, two unexplained deaths of the Yangtze sturgeon population occurred in Sichuan (on 8th July and 10th August, respectively; this study only obtained samples from the second incident), with mortality rates of 40.7% and 50.1%, respectively. The dying fish showed symptoms such as lethargy, loss of appetite, irregular swimming, anoxemia, and acute death. The disease outbreaks have had a significant impact on the health maintenance of the Yangtze sturgeon population in this base, and it is urgent to further study the etiology and develop targeted prevention and control strategies. Therefore, this study aims to elucidate the etiology and pathology of the disease outbreak, so as to provide theoretical support for the healthy breeding and disease prevention and control of Yangtze sturgeon, as well as provide reference for the protection of other endangered fish species.

## 2. Materials and Methods

### 2.1. Experimental Fish and Sample Collection

#### 2.1.1. Experimental Fish

**Figure 1:**
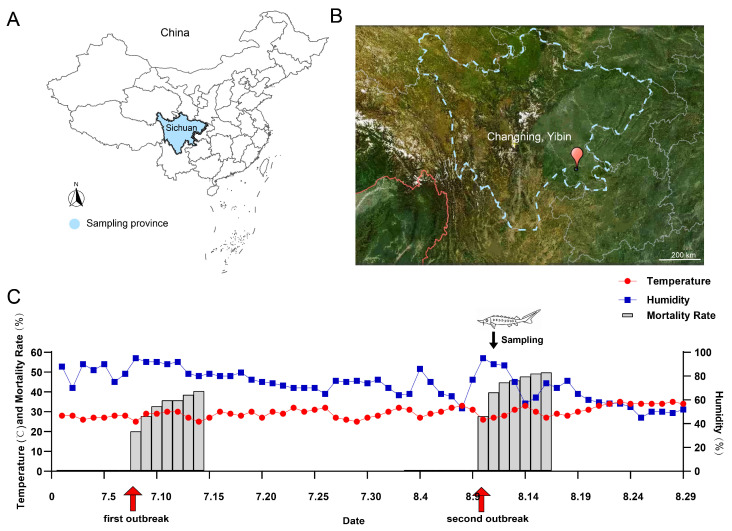
Sampling location sketch, meteorological data and the mortality rates. (**A**): The sampling province was located in Sichuan Province, China. (**B**): The samples were collected in Changning County, Yibin City, Sichuan Province. (**C**): Time series graph of temperature/humidity against fish mortality (including the mortality rate within seven days before and after each disease outbreak). (The temperature and humidity information is provided by Huiju Environmental http://www.hjhj-e.com/) Note: this study only obtained samples from the second outbreak.

The Yangtze sturgeon used in this study originated from the Changning Base of the Fisheries Research Institute of Sichuan Academy of Agricultural Sciences (Agriculture and Rural Affairs Ministry’s Protection Base for Rare and Endemic Fish Species in the Upper Reaches of the Yangtze River). The specific sampling locations are shown in Figure 1A,B, and the local temperature/humidity is shown in Figure 1C. The water used for the cultivation of sturgeon was recycled water (the water quality on the sampling day: dissolved oxygen: 5.7 mg/L; pH: 8.83; ammonia nitrogen: 1.07 mg/L; nitrite: 0.41 mg/L) And there were no potential pollution risk points such as farmland, industrial sites, and sewage outlets around the breeding base.

According to the Regulations on the Protection and Management of Aquatic Life in the Yangtze River (Order of the Ministry of Agriculture and Rural Affairs No. 5 of 2021) and the Law of the People’s Republic of China on the Protection of the Yangtze River (Order No. 65 of the President), raising endangered fish like Yangtze sturgeon in recirculatory water with ideal conditions is not subject to ethical review. All animals were treated in accordance with relevant guidelines and regulations given by the Animal Care and Use Committee of the Fisheries Research Institute, Sichuan Academy of Agricultural Sciences. The experimental protocols used for live fish experiment were based on the Animal Welfare Act.

#### 2.1.2. Sample Collection

A total of 6 fish were collected in this study, with 3 fish in each group (weight: 1018.34 ± 1.53 g, body length: 47.24 ± 0.47 cm). Prior to tissue sampling, the fish were anesthetized using a neutralized MS222 solution (Aladdin, Shanghai, China) at a final concentration of 200 mg/L. Subsequently, skin and gills from each fish were collected under sterile conditions. Direct Squash and microscopic observation were performed using a Nikon Eclipse E200 microscope (Tokyo, Japan) at 4× and 10×. No obvious parasites were found. Subsequently, ascites, skin ulcer lesions, and organs such as the heart and liver were collected under sterile conditions for bacterial isolation and identification. Additionally, the heart, liver, and kidney of the diseased fish were collected and fixed in 4% paraformaldehyde for histopathological analysis, and stored at −80 °C for molecular biological analysis. This study only obtained samples from the second outbreak.

### 2.2. Bacterial Isolation

The ascites, skin ulcers, liver and heart tissues collected were, respectively, inoculated into LB medium (Hu Kai Microbiology, Guangzhou, China, C28334B). The specific composition of the medium is as shown in Supplementary Table S1. After culturing in a constant temperature incubator at 30 °C for 48 h [8], the morphological characteristics of the bacteria were observed. Subsequently, the colonies of well-formed and distinct isolated colonies on the LB medium were further selected and re-inoculated on fresh LB medium, and then incubated in a constant temperature incubator at 30 °C for 24 h for purification. Ultimately, 12 single colonies were successfully obtained (3 fish × 4 tissues = 12 isolates).

### 2.3. Bacterial DNA Extraction and Amplification

Bacterial DNA extraction and purification were carried out using the isolation kit (Foregene, Chengdu, China) in accordance with the manufacturer’s instructions. The bacterial housekeeping gene (16S rRNA gene) was selected as the target for amplification, with specific primers 27F (5′-AGAGTTTGATCCTGGCTCAG-3′) and 1492R (5′-TACGGCTACCTTGTTACGACTT-3′) [9]. The PCR reaction system and thermal cycling conditions were set based on the methodologies established by Dunbar et al. [10].

### 2.4. Sequencing and Phylogenetic Tree Analysis

The PCR products were collected and submitted to Sangon Biotech (Shanghai, China) for purification and sequencing. The sequences were subjected to BLAST+ (v2.15.0) alignment in the NCBI database. Phylogenetic analysis was conducted using the mega software (v11.0) and the maximum likelihood (ML) method [11]

### 2.5. Quantification Determination of Bacterial Load

Specific primers were designed based on the conserved sequences of the target virulence genes of different bacteria (primer sequences and annealing temperatures are shown in Supplementary Table S2). Using the method established by Xue et al. [12], we quantitatively determined the bacterial load in liver, kidney, and heart tissues using the real-time fluorescence quantitative PCR system (Bio-Rad, Pleasanton, CA, USA). Briefly, standard curves were constructed by 10-fold serial dilution (10^9^–10^4^ copies/μL) of genomic DNA from pure cultures of each target bacterium, with copy numbers calculated based on DNA concentration (measured by Qubit) and bacterial genome size. Total DNA was extracted from liver, kidney, and heart tissues of healthy and diseased sturgeons (50 mg per sample) using a tissue Animal Tissue DNA Isolation Kit (Foregene, Chengdu, China), and adjusted to 10 ng/μL. The qPCR reaction system (20 μL) contained 10 μL SYBR Green qPCR Mix (with ROX reference dye), 0.8 μL of each primer (10 μM), 2 μL template DNA, and 6.4 μL nuclease-free water. Bacterial load (copies/g tissue) was calculated using the standard curve (R^2^ ≥ 0.99, amplification efficiency 90–105%) and normalized to tissue weight using the formula as follows:

Log_10_ (bacterial load, copies/g) = Log_10_ [(Copies/μL from qPCR) × (Total DNA elution volume, μL)/(Tissue sample weight, g)]

### 2.6. Staining

Two groups of heart, liver, and kidney samples fixed in 4% paraformaldehyde were paraffin-embedded. The paraffin-embedded eye samples were then sectioned using a Leica RM2235 microtome (Wetzlar, Hesse, Germany) to prepare tissue sections with a thickness of 5 µm. Subsequent to sectioning, the tissue sections were subjected to Hematoxylin-Eosin (H&E) Staining [13] and Gram Staining [14] in accordance with relevant methods, followed by sealing with neutral resin. Finally, the sealed tissue sections were observed and photographed under a Nikon Eclipse E200 (Nikon, Tokyo, Japan) with 4×, 40× and 100× objectives.

### 2.7. Transmission Electron Microscope (TEM) Observation

We selected the areas where the accumulation of bacteria was observed, melted the wax within that area and immersed it in pure acetone. Subsequently, the liver, kidney and heart tissues were washed with PBS, fixed with 1% osmic acid, dehydrated with gradient acetone, replaced and embedded. Then, the samples were stained with 1% uranyl acetate for 20 min, rinsed with ddH_2_O twice, followed by staining with 1% lead citrate for 20 min, and another two washes with ddH_2_O. Finally, the ultrastructure of the bacteria was observed by HITACH type I transmission electron microscope under 80 kV. Images were captured and collected for subsequent analysis.

### 2.8. Antibiotic Sensitive Test

The Kirby–Bauer disk diffusion method was used to assess the sensitivity of isolated strains to 14 common antibiotics. Strains were cultured in LB broth at 30 °C for 24 h, adjusted to 1.5 × 10^8^ CFU/mL (0.5 McFarland standard) in sterile PBS, and uniformly swabbed onto LB plates. Antibiotic disks (Oxoid Ltd., Basingstoke, Hampshire, UK) were aseptically placed, and plates were incubated at 30 °C for 48 h. Inhibition zones were measured with a vernier caliper, and antibiotic sensitivity was regarded as susceptible (S), intermediate (I), or resistant (R) according to the Clinical and Laboratory Standards Institute (CLSI) guidelines (M100-S26, 2016) [15]. All tests were conducted in triplicate, and *Escherichia coli* ATCC 25922 was the control strain.

### 2.9. Data Analysis and Statistics

In this study, all data were presented as mean ± SD (standard deviation). Charts were drawn by GraphPad Prism (v8.0, GraphPad Software, Inc., San Diego, CA, USA) and Adobe lllustrator (v 28.7.8, Adobe Inc., San Jose, CA, USA). The experimental data were first subjected to normality tests. Then, independent sample *t*-tests were used to evaluate the significance of the differences, and the significance level was set as *p* < 0.05.

## 3. Results

### 3.1. Clinical Symptoms of Infected Yangtze Sturgeon

**Figure 2:**
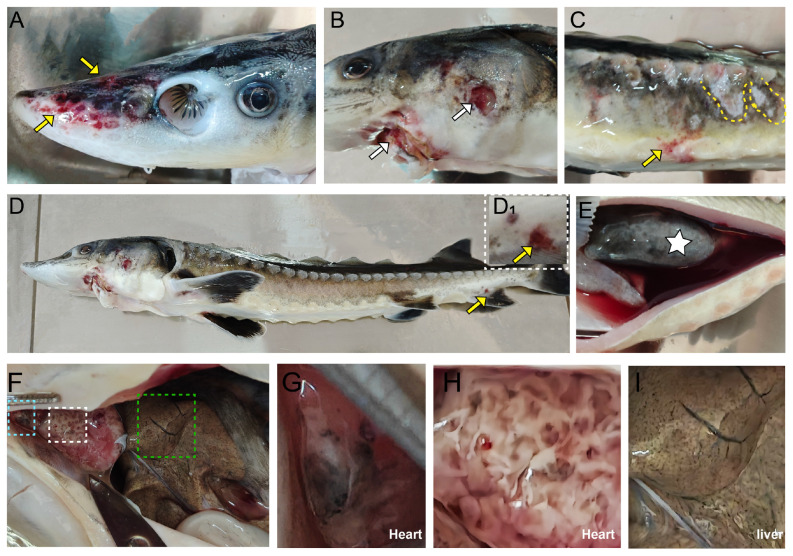
Gross clinical symptoms. (**A**): Hemorrhaged at the rostral side (yellow arrow). (**B**): Ulcer (white arrow). (**C**): Hemorrhaged at the body surface (yellow arrow) and ulcerative lesions (dotted line). (**D**): Clinical symptoms of the diseased Yangtze sturgeon. (**D_1_**), Hemorrhaged at the vent (yellow arrow). (**E**): Upon dissection, the swim bladder appeared darkened (star), accompanied by copious hemorrhagic ascites. (**F**): Lesions of internal organs, black bulbus arteriosus (blue dotted line), uneven white cystic mass in the epicardium (white dotted line), and discrete dark punctate lesions in the liver (green dotted line). (**G**–**I**): The enlarged view of the three different colored dotted lines in (**F**).

The infected sturgeon exhibited multi-focal tissue lesions throughout the bodies, manifesting as severe hemorrhagic septicemia. The main pathological changes were as follows: congestion and hemorrhage at the rostrum, body surface and vent (Figure 2A–D), body surface ulcers (Figure 2B) and skin ulceration (Figure 2C). Dissection revealed that the diseased sturgeon exhibited a darkened swim bladder accompanied by substantial hemorrhagic ascites (Figure 2E). Furthermore, the samples manifested significant internal organ lesions (Figure 2F). These lesions included a dark-colored heart, dark spots on the bulbus arteriosus (Figure 2G), irregular white cyst-like lumps on the epicardium (Figure 2H), and small separate dark dot-like lesions in the liver (Figure 2I).

### 3.2. Isolation and Identification of Pathogens

**Figure 3:**
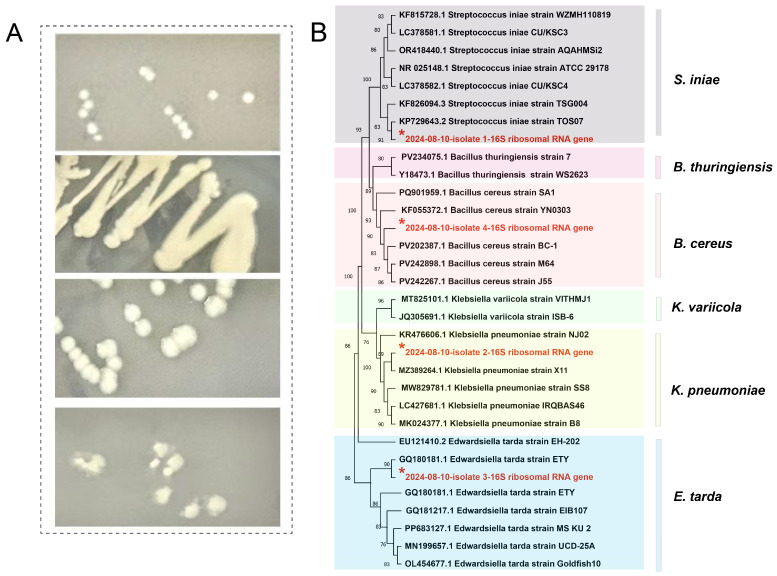
Bacterial morphology and phylogenetic tree analysis. (**A**): Four types of bacteria with different morphologies were isolated from the infected sturgeon. (**B**): Phylogenetic tree based on the 16sr RNA gene and the maximum likelihood method with bootstrap values from 1000 replicates. The red asterisks indicate specific 16S rRNA gene isolates. Note: The 2nd isolated strain is homologous to the giant panda *K. pneumoniae* strain (MZ389264.1), and this is the first report of the association of this cross-species strain. However, the phylogenetic relatedness does not necessarily prove transmission pathways.

After bacterial isolation and purification, these 12 isolates were finally categorized into 4 distinct pathogenic species (not all 4 species were present in every fish). The specific morphological features of these species are as follows: small-diameter, grayish-white circular colonies; large-diameter, mucinous colonies; medium-diameter, semi-transparent, flat-round colonies; and medium-diameter, rough-surfaced colonies (Figure 3A). For species confirmation, the full-length 16S rRNA gene sequences of the four representative isolates were amplified and sequenced, with sequence lengths ranging from 1445 to 1501 bp (covering the V1–V9 hypervariable regions). Detailed sequence alignment parameters are shown in Table S3. The phylogenetic tree analysis constructed based on the 16S rRNA gene sequence showed that these four isolates formed significant clusters with *Streptococcus iniae*, *Klebsiella pneumoniae*, *E. tarda*, and *Bacillus cereus*, respectively (Figure 3B).

### 3.3. Histology Observation

To analyze the pathological damage of sturgeon caused by the bacterial co-infection, H&E staining and Gram staining were conducted.

**Figure 4:**
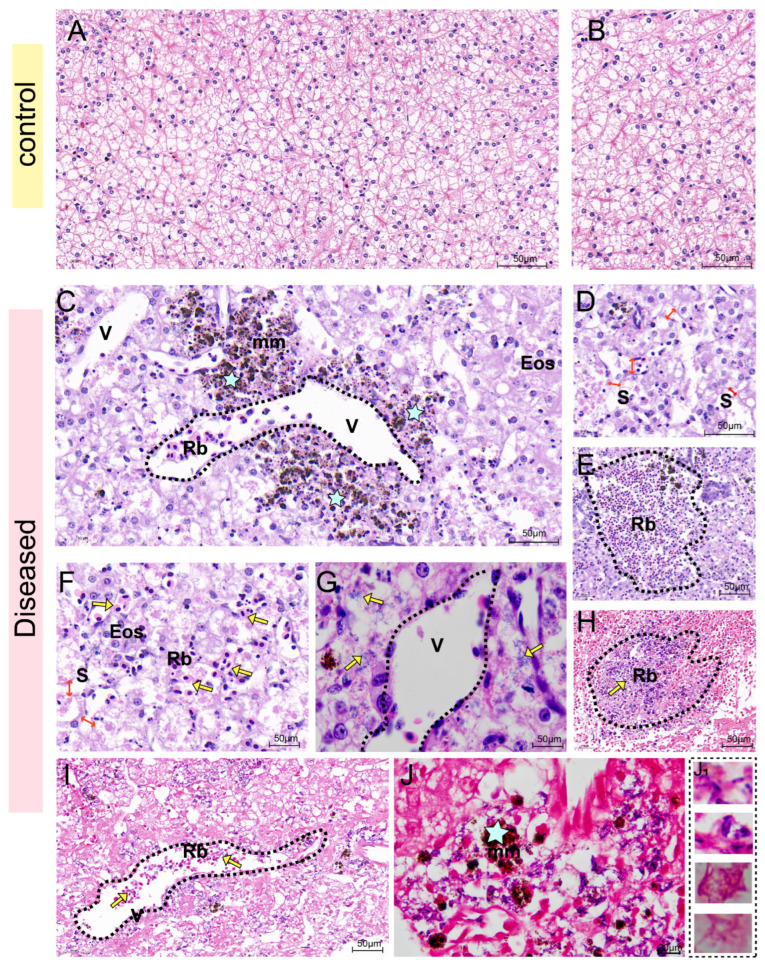
H&E staining and Gram staining of the liver. (**A**,**B**): H&E staining of healthy sturgeon. (**C**–**G**): H&E staining of diseased sturgeon. (**C**): A large number of melanin macrophages (star) were aggregated around the central vein (dotted line) of the liver. Red blood cell exuded in the central vein presents as vasculitis. Plenty of hepatocytes show vacuolization and necrosis. (**D**): The space between hepatic sinusoids was dilated (red double—headed arrow), and the intercellular substance was necrotic. (**E**): Exudated red blood cells aggregated. (**F**): Abundant red blood cell exudation in the intercellular substance (yellow arrow). (**G**): Abundant bacteria (yellow arrow) and eosinophils around the central vein (dotted line). (**H**–**J**): Gram staining. (**H**): A large number of bacteria (yellow arrow) presented around the exuded red blood cells (dotted line). (**I**): Red blood cell exudation and bacterial infection (yellow arrow) in the central vein of the liver (dotted line). (**J**): The bacteria presented different morphological characteristics. (**J_1_**): Gram-positive bacteria and Gram-negative bacteria had different morphological characteristics. Note: Previous reports mostly focused on the presence of single bacterial aggregations within liver tissues. This study, however, observed the co-localization of multiple Gram-positive/Gram-negative bacteria. V, central vein; mm, melanin macrophage; Rb, erythrocyte; Eos, eosinophils; S, hepatic sinuses.

From the H&E staining results of the liver, the hepatocytes of healthy sturgeon were neatly arranged in a cord-like pattern, with prominent nucleoli and abundant hepatic sinusoids (Figure 4A,B). In the infected sturgeon liver, obvious red blood cell exudation was seen in the hepatic central vein, manifesting as distinct vasculitis. Additionally, a large number of melanin macrophages and eosinophils aggregated around the central vein (Figure 4C). Many hepatocytes showed vacuolation or necrosis (Figure 4C), and the spaces between hepatic sinusoids were dilated (Figure 4D). Abundant exuded red blood cells were present in the intercellular substance (Figure 4E,F), and a large number of bacteria around the central vein (Figure 4G). Gram staining provides a clearer view, showing that there were a large number of bacteria surrounding the exuded red blood cells (Figure 4H) and in the central vein of the liver (Figure 4I), which displayed diverse morphological characteristics (Figure 4J).

**Figure 5:**
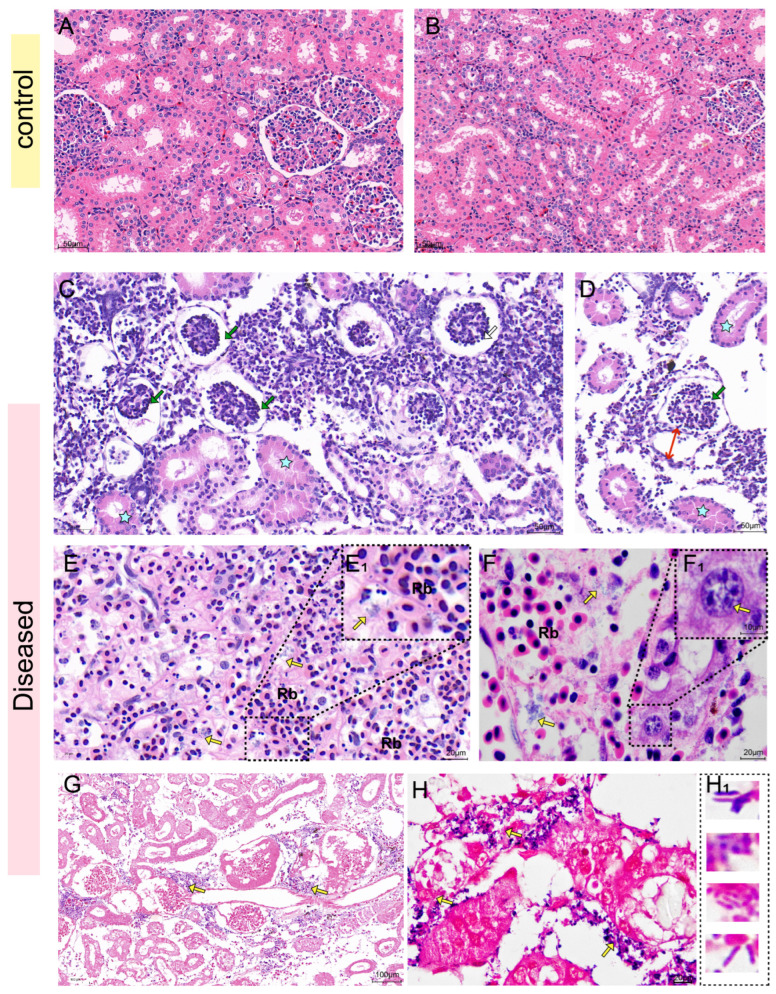
H&E staining and Gram staining of the kidney. (**A**,**B**): H&E staining of healthy sturgeon. showing intact renal corpuscle and neatly arranged epithelial cells of the renal tubules. (**C**–**H**): H&E staining of diseased sturgeon. (**C**): Glomerular atrophy (green arrow), red staining in the cytoplasm of renal tubular epithelial cells (star). (**D**): Dilated space between renal tubules, and dilated renal capsule (red double—headed arrow). (**E**): There was large amount of red blood cell exudation in the intercellular substance with abundant short-rod-shaped bacteria around (yellow arrow). (**E_1_**): Local magnification of the black dotted line in (**E**,**F**): Abundant bacteria (yellow arrow) could be seen in intercellular substance. (**F_1_**): Local magnification of the black dashed line in (**F**), showing rod-shaped bacteria in macrophages (yellow arrow). (**G**,**H**): Gram staining: (**G**): Abundant bacteria in kidney tissue (yellow arrow). (**H**): The bacteria (yellow arrow) presented different morphological characteristics. (**H_1_**): Gram-positive bacteria and Gram-negative bacteria had different morphological characteristics. Note: Previous reports mostly focused on the presence of single bacterial aggregations within kidney tissues. This study, however, observed the co-localization of multiple Gram-positive/Gram-negative bacteria. Rb, red blood cells.

According to the H&E staining results of the kidney, it can be seen that the renal corpuscle of the healthy sturgeon was intact, the glomerulus and renal capsule were clear, and the renal tubular epithelial cells are neatly arranged (Figure 5A,B). However, the infected sturgeon exhibited obvious pathological changes, including glomerular atrophy and degeneration and necrosis of renal tubular epithelial cells (Figure 5C), along with loose dilation of the spaces between renal tubules and obvious dilation of the renal capsule (Figure 5D). Moreover, there was an abundance of exuded proteins in the tubular lumens, resulting in obvious eosinophilic staining of the cytoplasm of renal tubular epithelial cells (Figure 5C,D). In addition, a large number of red blood cells had exuded into the intercellular substance, which was surrounded by numerous bacteria (Figure 5E,F). Gram staining revealed the presence of a large number of both Gram-positive and Gram-negative bacteria in the kidney tissue (Figure 5G). These bacteria exhibited diverse morphological characteristics, with some being spherical and others rod-shaped (Figure 5H).

**Figure 6:**
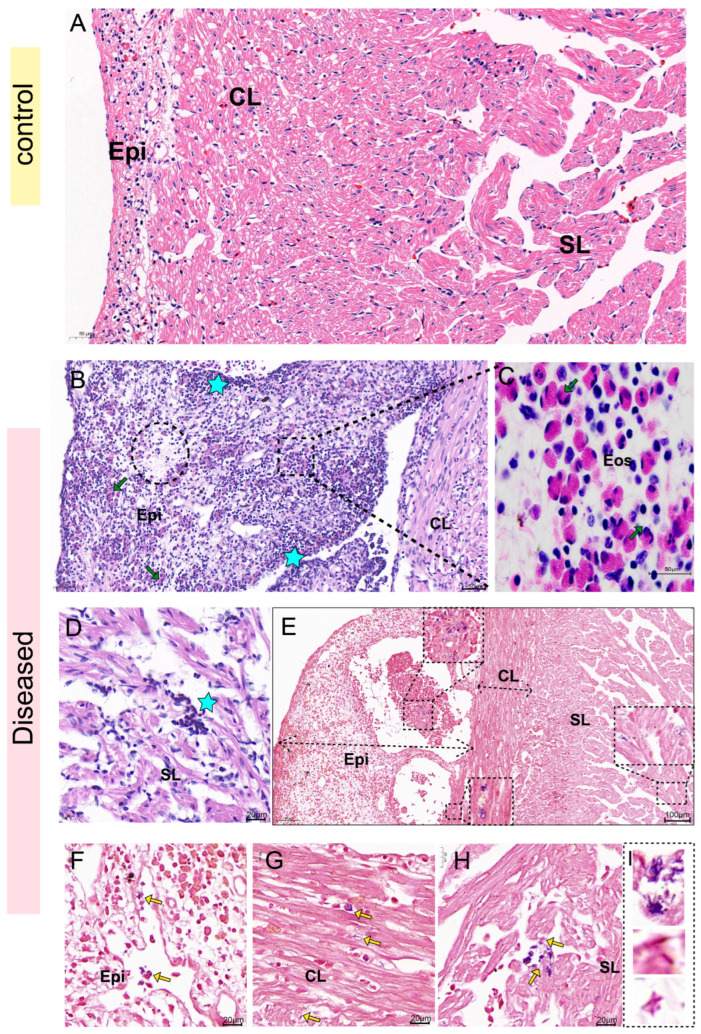
H&E staining and Gram staining of the heart. (**A**): H&E staining of healthy sturgeon. (**B**–**D**): H&E staining of diseased sturgeon. (**B**): The epicardium thickened, with abundant lymphocyte infiltrated (star). Eosinophils clustered (green arrow), with severe cell vacuolation and necrosis (dotted circles). (**C**): Abundant eosinophils (green arrow). (**D**): Myocarditis, lymphocyte infiltration in the spongy layer (star). (**E**–**I**): Gram staining: (**E**): Bacteria could be seen in the epicardial layer, compact layer, and spongy layer of the heart (dashed line box). (**F**–**H**): Bacteria (yellow arrow) in the epicardial layer, compact layer, and spongy layer respectively. (**I**): Gram-positive bacteria and Gram-negative bacteria had different morphological characteristics. Note: Previous reports mostly focused on the presence of single bacterial aggregations within heart tissues. This study, however, observed the co-localization of multiple Gram-positive/Gram-negative bacteria. Eos, eosinophils; Epi, epicardial layer; CL, compact layer; SL, spongy layer.

The H&E staining results of the heart showed that the three-layer membrane structure of the heart of healthy sturgeon was intact, the myocardial cells were neatly arranged with clear transverse stripes, and there was no infiltration of inflammatory cells (Figure 6A). However, the epicardium of the affected sturgeon was thickened, with severe cell vacuolation and necrosis (Figure 6B). Moreover, a substantial number of lymphocytes infiltrated, and eosinophils aggregated within the epicardial layer, compact layer, and spongy layer of the heart (Figure 6C,D). Gram staining showed that both Gram-positive and Gram-negative bacteria were found in the epicardial layer, compact layer and spongy layer of the heart tissue of the diseased fish (Figure 6E–I), but the quantity of bacteria was slightly lower compared to that in the liver and kidney.

### 3.4. Ultrastructural Analysis

**Figure 7:**
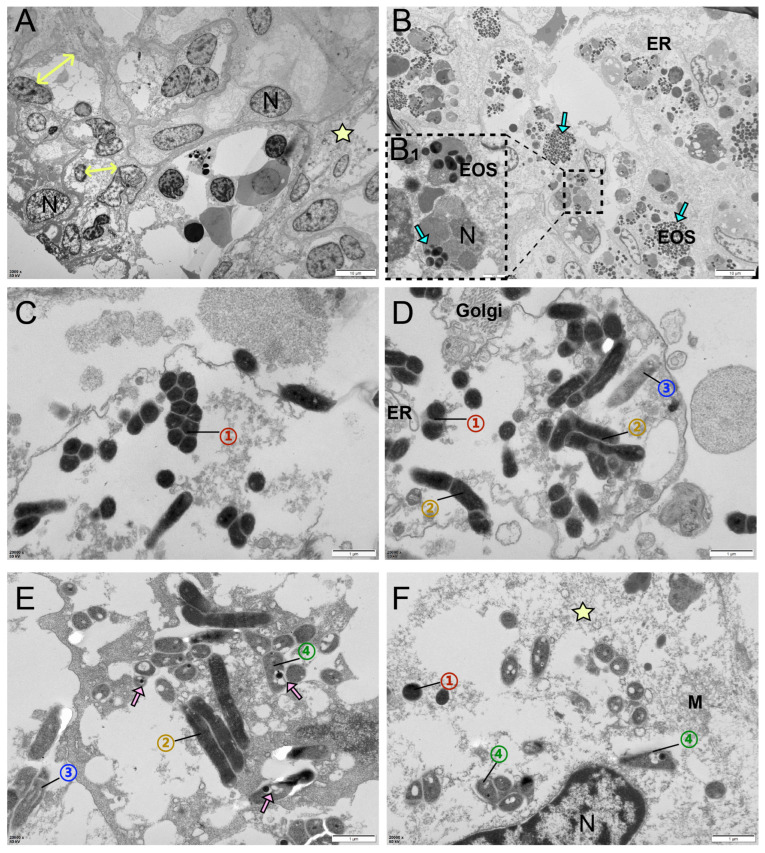
TEM observations. (**A**): Pyknosis of renal cell nuclei and dilation of epithelial cells (yellow double—headed arrow), lymphocyte infiltration, and diffuse necrosis (star). (**B**): Extensive eosinophil infiltration (blue arrow), (**B_1_**) showing eosinophils invading nuclear interiors. (**C**–**F**): Pathogenic morphological features of bacterial co-infection. (**C**): Dark spherical bacteria arranged in chains (①). (**D**): Dark spherical bacteria (①), paired or solitary dark bacilli (②), and light-colored bacilli (③) observed in the field of view. (**E**): The fourth bacterial morphotype (④) with low electron density and intracellular endospores (pink arrow). (**F**): The pathogenic bacteria were varied in morphology. Diffuse necrosis (star) and mitochondrial disintegration. Eos, eosinophils; N, nucleus; ER, endoplasmic reticulum; M, mitochondria; Golgi, Golgi apparatus.

TEM examinations were conducted on the kidneys, livers and hearts of the diseased sturgeon, and no viral inclusions or parasites were detected. However, it was observed that a variety of bacteria had extensively colonized each tissue of the infected sturgeon, resulting in diffuse necrosis, lymphocyte infiltration, and cellular vacuolation, and severe disruption of organelle integrity was observed in all tissues (Figure 7A). Notably, eosinophil infiltration was evident not only in the interstitial spaces but also within the nuclear interiors of affected cells (Figure 7B). In addition, through morphological characterization, four distinct bacterial morphotypes were identified. These included chain-forming dark spherical bacteria (Figure 7C), paired or solitary dark bacilli (Figure 7D,E), solitary light-colored bacilli (Figure 7D,E), and light-colored bacilli containing intracellular endospores (Figure 7E,F).

### 3.5. Bacterial Load and Antibiotic Susceptibility

**Figure 8:**
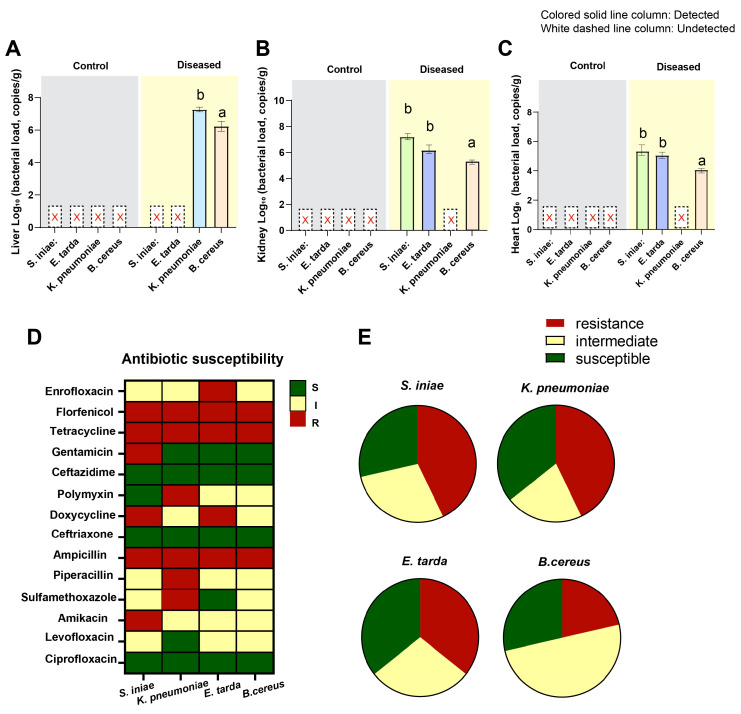
Analysis of bacterial load and antibiotic susceptibility tests. (**A**–**C**) The bacterial load in liver, kidney, and heart tissues, respectively. The white dashed columns indicate undetected, while the differently colored solid columns represent the detection of different bacteria. The significance level was set at *p* < 0.05. Among them, ‘a’ and ‘b’ were used to indicate significant differences, while groups marked with the same lowercase letter had no significant differences among them, and groups marked with different lowercase letters had significant differences. (**D**) Antibiotic susceptibility heat map, green, yellow and red represent sensitive, intermediate and resistant, respectively. (**E**) Proportion pie graph of pathogen susceptibility, showing the distribution of “resistant/intermediate/sensitive” proportions of the pathogen to 14 tested antibiotics.

By conducting bacterial load detection on liver (Figure 8A), kidney (Figure 8B) and heart (Figure 8C) tissues, it was found that no bacteria were detected in the control group. The liver of the diseased sturgeon was found to contain *K. pneumoniae* and *B. cereus*, the kidneys tested positive for *S. iniae*, *E. tarda*, and *B. cereus*. And the heart was found to have *S. iniae*, *E. tarda*, and *B. cereus*. Based on the analysis of the experimental results of antibiotic sensitivity tests, it was found that the antibiotic resistance of these four strains showed significant species differences. But at the same time, there were also common characteristics: All showed a highly sensitive state to ceftazidime, ceftriaxone and ciprofloxacin, while showing significant resistance to florfenicol, tetracycline and ampicillin (Figure 8D). In addition, the antibiotic resistance of *S. iniae*, and *K. pneumoniae* was the strongest, followed by, *E. tarda*. *B. cereus* had the weakest MDR and was more often in an “intermediate sensitivity” state to most antibiotics (Figure 8E).

## 4. Discussion

### 4.1. The Transition from Single Pathogen to a Multi-Pathogen Disease Pattern

During the second outbreak in 2024, four types of bacteria were isolated from the tissues of diseased Yangtze sturgeon: *S. iniae*, *K. pneumoniae*, *E. tarda*, and *B. cereus*. Through histopathological and ultrastructural pathological analyses, the co-localization of these four bacteria in the necrotic lesions was determined. Additionally, bacterial load analysis indicated that all four pathogenic bacteria were not only present in the lesion tissues, but their detection rates were highly consistent with the severe clinical symptoms exhibited by the diseased sturgeons (hemorrhagic septicemia, organ necrosis, acute death). These findings collectively suggest a strong association between these four bacterial species and the disease outbreak.

Furthermore, our research revealed the phenomenon of severe polymicrobial infection in the Yangtze sturgeon, marking a significant shift in the traditional understanding of the Yangtze sturgeon’s bacterial ecology. Previous studies mostly documented pathological and physiological conditions related to a single pathogen, such as *A. hydrophila* and *E. tarda* [4,5]. Although these studies were very valuable, they essentially focused on one-on-one “host–pathogen” conflicts. We discovered a co-infection involving *S. iniae*, *K. pneumoniae*, *E. tarda*, and *B. cereus*, which provides a more complex and potentially more accurate explanation for the observed acute, multi-systemic damage.

### 4.2. The Isolated Strains in This Study Pose a Serious Threat to Aquatic Ecosystems

The four bacteria isolated in this study are not only significant pathogens in aquaculture but also zoonotic agents [16]. They frequently trigger sudden disease outbreaks in farmed fish, posing a serious threat to the aquatic ecosystems. Therefore, appropriate measures must be implemented to prevent and control the infection and spread of these pathogenic bacteria [17,18].

Among the identified pathogenic bacteria, *S.iniae* is an important primary pathogen that can infect a variety of wild and farmed fish. Due to its high pathogenicity and fatality rate, it has caused huge economic losses to the global aquaculture industry [19]. Although *S. iniae* typically induces chronic infections in fish (such as exophthalmia or corneal opacity) [20], it can also provoke acute manifestations such as acute septicemia [21,22]. It has also been reported to be susceptible to cefotaxime, erythromycin, ofloxacin, penicillin, and vancomycin, but resistant to florfenicol [23]. In the present study, infected sturgeon exhibited acute systemic symptoms, including ulcerative skin lesions, hemorrhagic septicemia, diffuse organ necrosis, and acute death. Thus, under high-temperature stress, *S. iniae* likely triggered acute infection in this species.

Notably, *K. pneumoniae* [24], *E. tarda* [25], and *B. cereus* [26] are opportunistic pathogens in aquaculture, capable of causing severe disease under compromised host conditions. Recently, *K. pneumoniae* has been proven capable of infecting a variety of aquatic animals, such as causing renal necrosis, structural changes and tubular swelling in *Labeo rohita* [27], as well as hepatocyte vacuolization and systemic infection in *Amphiprion nigripes* [28]. Moreover, it has been reported to be highly resistant to carbapenem antibiotics [24]. *E. tarda* triggers Edwardsiellosis, which is usually associated with sudden outbreaks and high mortality rates of fish diseases, leading to symptoms such as surface ulcers, visceral necrosis and systemic infections [17]. It has also been reported to be sensitive to chloramphenicol, ciprofloxacin, and streptomycin, but resistant to amoxicillin, erythromycin, and flumequine [29]. In recent years, although the application of *B. cereus* as a probiotic has also witnessed a gradual upsurge [30], its pathogenic potential has been confirmed: this bacterium can be isolated from the internal organs or lesions of diseased fish, causing hemorrhagic septicemia, skin ulcers, and red head in fish [31]. Furthermore, reports on the antibiotic sensitivity of its pathogenic strains remain relatively scarce. In this study, all three species co-occurred in diseased sturgeon, manifesting hemorrhagic septicemia, cutaneous ulcers, and systemic infections. This consistent with their opportunistic nature where environmental stress or host immunodeficiency enables virulence.

Histopathological examination in this study revealed co-localization of *S. iniae* with opportunistic pathogens within necrotic lesions. Collectively, these observations lead us to hypothesize that: *S. iniae* first exploited the mucosal immune system damaged by high temperatures, leading to micro-ulcers and vascular damage, thereby facilitating systemic dissemination. Subsequently, some opportunistic pathogens (such as *K. pneumoniae*) colonized in the damaged tissues via bacterial synergism, exacerbating the lesion. However, it should be noted that due to the lack of bacterial load analysis during the early stage of the disease, the chronological sequence hypothesis proposed in this study is merely a speculation based on the existing indirect evidence, rather than an absolute conclusion.

### 4.3. Possible Causes of the Disease Outbreak

The disease outbreak coincided with severe environmental deterioration, which critically increased the host’s susceptibility to infection. As a cold-water species, the Yangtze sturgeon has an optimal growth temperature of 20–22 °C [32]. The persistently high water- temperature of approximately 30 °C during the outbreak subjected the fish to intense thermal stress, disrupting internal homeostasis and causing dysregulation of the immune system. This physiological compromise significantly heightened the host’s vulnerability to pathogens. From an ecological perspective, the same elevated temperature provided ideal thermodynamic conditions for the rapid proliferation of the four mesophilic bacterial pathogens isolated in this study [33]. effectively shifting the host–pathogen balance in favor of the latter.

Furthermore, the high temperature is likely to have accelerated the decomposition of the feed, leading to eutrophication and deterioration of water quality. As the water quality data on the sampling day showed (dissolved oxygen: 5.7 mg/L, pH: 8.83, ammonia nitrogen: 1.07 mg/L, nitrite: 0.41 mg/L), it clearly indicates that the environmental conditions have seriously deteriorated, which in turn promotes the reproduction and adaptation of bacteria.

Moreover, high humidity during the outbreak weakened the protective function of the sturgeon’s body surface mucous layer, reducing its viscosity and antibacterial activity, which in turn facilitated bacterial attachment and invasion. Simultaneously, the high-humidity environment favored bacterial survival, reproduction, and virulence. It also expanded the pathogens’ transmission routes (such as diffusion via water flow or aerosol formation) that enables respiratory invasion [34], which greatly aggravates the complexity of the disease.

Considering the high pathogenicity of *S. iniae* and the opportunistic potential of *K. pneumoniae*, *E. tarda*, and *B. cereus* [35], we hypothesize that the high-temperature and high-humidity during the summer were the key factors contributing to water quality deterioration and the outbreak of this disease. It was precisely the deterioration of environmental conditions and the reduced immunity of the host that enabled the bacteria already existing in the aquaculture environment to multiply and trigger serious infections.

### 4.4. The Risk of Cross-Host–Pathogen Transmission in the Aquaculture Industry

In this study, phylogenetic analysis revealed that the *S. iniae* isolate from this research clustered with the diseased *Trachinotus ovatus* (golden pomfret) strain (KP729643.2) from Zhanjiang, Guangdong Province, and exhibited a high degree of homology. This suggests potential cross-species transmission risks of pathogens between the Yangtze sturgeon and marine aquaculture fish species. Within the interaction between southern coastal mariculture and inland freshwater aquaculture in China, frequent seed transportation is often accompanied by the migration of live carriers harboring pathogens, while feed may serve as vectors for pathogen attachment and dissemination during production, transportation, and feeding processes. These factors collectively establish potential pathways for cross-host transmission of *S. iniae*, posing a threat to the diverse cultured species within the aquaculture industry.

The *K. pneumoniae* isolate identified in this study demonstrated high homology to a strain (MZ389264.1) originating from Chengdu Giant Panda Breeding Station. Although there is no direct water connection between the two bases, residual *K. pneumoniae* in water and soil within giant panda habitats may undergo natural dispersal via tributaries of the Yangtze River system and agricultural irrigation networks of the Sichuan Basin (such as the Min River-Dujiangyan water system), consequently manifesting as clustering on the phylogenetic tree. This phenomenon not only reflects the bacterium’s robust environmental adaptability and dispersal capacity but also highlights the potential for cross-transmission of pathogens between aquaculture environments and terrestrial wildlife habitats.

The *E. tarda* isolate in this study shared high homology with a strain (GQ180181.1) isolated from *eel* in Fuzhou, Fujian Province. This is presumably attributed to the cross-provincial circulation of seedlings (such as introduction of *eel* fry into Sichuan) and exchanges of aquaculture technologies in China’s freshwater aquaculture industry. Such cross-provincial seedling circulation enables pathogens to traverse geographical barriers alongside their hosts. Moreover, If the tools and equipment used in the exchange of aquaculture techniques are not thoroughly sterilized, they may also act as transmission vectors. Consequently, *E. tarda* maintains its potential for transmission and pathogenicity across diverse hosts and geographical regions.

However, it must be clearly emphasized that phylogenetic relatedness only reflects correlations in strain distribution and does not equate to a clearly demonstrated transmission path. Future work should prioritize the use of advanced techniques such as whole-genome sequencing to conduct in-depth investigations into potential genetic differences between different strains. Concurrently, it is essential to analyze the molecular mechanisms underlying host specificity and the evolutionary characteristics of these strains, thereby enhancing the ability to interpret the transmission chain.

### 4.5. The Antibiotic Resistance (AMR)

Antibiotic treatment represents one of the primary measures for disease prevention and control in aquaculture industry. However, AMR has become an important threat to the sustainable development of the global aquaculture industry. Due to the intrinsic connection between aquaculture systems and open water bodies such as rivers and lakes, antimicrobial use (AMU) in aquaculture has greater potential for environmental transmission compared to terrestrial animals, thereby having a more significant impact on ecosystems and public health [36]. In high-density intensive aquaculture, although the preventive use and therapeutic abuse of antibiotics aim to prevent and treat bacterial infections in fish, they form a vicious cycle of “drug use—evolution of drug resistance” in the aquaculture environment [37]. The current spread of drug resistance genes (ARGs) caused by the abuse of antibiotics not only intensifies the difficulty of disease prevention and control, but also promotes the horizontal transfer of drug resistance genes in the flora, and threatens public health and ecological security through the food chain and environmental pollution [38].

Our antimicrobial susceptibility testing revealed that the isolated *S. iniae*, *K. pneumoniae*, *E. tarda*, and *B. cereus* strains all exhibited MDR, particularly to florfenicol, tetracycline, and ampicillin, which are empirically and frequently used in clinical aquaculture [39], even when the specific pathogen type or drug sensitivity results are unclear [40]. This MDR pattern aligns with the historical and widespread use of these antibiotics in Chinese aquaculture, as evidenced by high detection rates of residues in aquatic products [41]. These findings underscore the urgent need to adopt a “One Health” approach to AMR management in aquaculture.

### 4.6. Recommendations for Disease Prevention, Management, and Conservation

Notably, a critical gap exists between in vitro lab efficacy and real-world application feasibility of the sensitive antibiotics we identified. While all four pathogens showed high sensitivity to ceftazidime, ceftriaxone, and ciprofloxacin in lab tests, China’s veterinary drug regulations explicitly prohibit the use of the first two in aquaculture. This restriction rendered only ciprofloxacin hydrochloride and berberine hydrochloride premix as viable therapeutic options for the 2024 outbreak.

For a Class I protected species like the Yangtze sturgeon, strict adherence to veterinary drug regulations is non-negotiable, as any unapproved antibiotic use could introduce toxic drug or ARGs into wild populations via stock enhancement programs, endangering the species’ long-term recovery. Consequently, there is an urgent need to prioritize non-antibiotic, preventive strategies aligned with the “One Health” principle. 

The following comprehensive measures are recommended: First, conduct proactive monitoring of meteorological trends to anticipate thermal stress events; second, use shading facilities or automated cooling systems to alleviate heat stress; third, strictly disinfect the circulating water (for example, through ultraviolet irradiation) to limit pathogen persistence; fourth, develop Chinese herbal medicine alternative therapies and precise vaccine control [42]. Such approaches are essential to minimize antibiotic reliance and mitigate the dissemination of ARGs.

### 4.7. The Limitations of This Study and the Future Directions

Regrettably, as an emergency field investigation addressing the Yangtze sturgeon bacterial disease outbreak, this study has notable limitations that need to be clearly pointed out. First, in terms of sample representativeness: two unexplained mortality events occurred in the Yangtze sturgeon population in Sichuan between July and August 2024 (with similar clinical symptoms). However, access to samples was only granted during the second outbreak. Consequently, our analysis is based solely on the samples collected from that single event, which may affect the generalizability of the findings. We cannot confirm whether the polymicrobial co-infection identified herein (the second outbreak) is consistent with the etiology of the first outbreak, nor can we rule out the potential variations in virulence gene expression or host–pathogen interaction patterns that may have existed between the two events.

Second, legal and ethical regulations protecting this critically endangered species precluded infection experiments to fulfill Koch’s postulates. Consequently, we cannot establish a definitive causal link or infection chronology. Therefore, the proposed roles of the isolated bacteria in the disease outbreak should be interpreted as strong correlations inferred from multiple lines of evidence, rather than proven causality.

Third, our pathogen analysis relied solely on culturable bacteria (the four isolated species bacteria) and lacked metagenomic or NGS-based profiling. This approach failed to capture non-culturable bacteria, low-abundance pathogens, or potential co-existing viruses and fungi, which limits ecological interpretation.

To address these limitations and improve the applicability of future research, our follow-up work will prioritize three directions: First, conduct metagenomic or NGS-based profiling to capture the complete pathogen community and clarify whether these uncaptured microbes interact with the four isolated bacteria to exacerbate disease. Second, select healthy Siberian sturgeon (*Acipenser baerii*) a non-protected species closely related to the Yangtze sturgeon) to conduct a time-series mixed infection study using the four pathogenic bacteria isolated in the original research, to verify the chronological sequence. Third, select the liver and kidney organs for transcriptome analysis, and conduct correlation analysis between the expression levels of DEGs, the bacterial load and the degree of tissue pathological damage, to clarify the association pattern between “pathogen bacterial load, host immune response, and pathological damage”.

Despite these limitations, the strong correlative evidence obtained in this study, including pathogen co-localization in lesions, bacterial load detection, and alignment with clinical symptoms, still provide valuable insights into the potential causes of the epidemic outbreak. Our study provides the first evidence of the four-pathogen co-infection in Yangtze sturgeon, and indicates that their presence is related to the environmental stress of high temperature and high humidity, offering important reference for emergency disease control and ecological conservation of this critically endangered species.

## 5. Conclusions

This study investigated the lethal disease outbreak in the endangered Yangtze sturgeon in the summer of 2024. The findings revealed that the bacterial co-infection (*S. iniae*, *K. pneumoniae*, *E. tarda*, and *B. cereus*) triggered by high temperature and high humidity environmental stress was closely related to this large-scale death event. Moreover, the isolated strains generally exhibited MDR, highlighting the urgency of implementing strict antibiotic management regulations in current aquaculture practices. Our research findings provide insights into the ecological prevention of endangered fish species, emphasizing the significance of transitioning from a single-pathogen perspective to a multi-pathogen framework. They also highlight the necessity of strengthening cross-regional pathogen monitoring and adopting non-antibiotic health management methods.

## Data Availability

Data will be made available on request.

## Supplementary Materials

The following supporting information can be downloaded at: https://www.mdpi.com/article/10.3390/biology14111498/s1, Table S1. LB agar plate culture medium formula component. Table S2. Primers sequence. Table S3. Sequencing and alignment parameters of 16s rrna genes for 4 pathogenic bacteria.
