# Supplementary material for: Evidence of Bacterial Co-Infection in Endangered Yangtze Sturgeon (Acipenser dabryanus)
Source: Biology (Basel). 2025 Oct 27;14(11):1498. doi: 10.3390/biology14111498 (PMC12650071; doi:10.3390/biology14111498)
Supplement: Supplementary file 1 [file biology-14-01498-s001.zip › biology-3882037-supplementary.pdf]

1. The specific formula components of the culture medium used in the research

Table S1. LB agar plate culture medium formula component

Formula /L	Content
Peptone	10.0g
Yeast extract	5.0g
Sodium chloride	5.0g
Agar	15.0g
Pure water	1000mL
pH 7.3±0.2	

2. The primers used for the detection of virulence genes in this study

Table S2. Primers sequence

Gene name	primer sequence (5′-3′)	Annealing temperature	GenBank accession number
simA	F:CCAAGATTTGGTTTTTGGATGTCG<br>R: TGCTTGTGCTTTCTCAAGGTC	56℃	MZ913320.1
rmpA	F:ACTGGGCTACCTCTGCTTCA<br>R: CTTGCATGAGCCATCTTTCA	57℃	KF801503.1
esrB1	F:GATCATGCCTTGCTAGCC<br>R: TCGGCGACCAGCTTGAGA	56℃	KM267087.1
nheA	F:GGAGGGGCAAACAGAAGTGAA<br>R: CGAAGAGCTGCTTCTCTCGT	57℃	PQ729833.1

3. The length of gene fragments, comparison parameters, and consistency/coverage data for bacterial identification

Table S3. Sequencing and alignment parameters of 16s rrna genes for 4 pathogenic bacteria

pathogenic bacteria	Query Sequence Length (bp)	Accessi on Length (bp)	Query Cover (%)	Percentage of Identity (%)	The most similar strain (Accession number)
S.iniae	1501	1512	99.2	99.5	*Streptococcus iniae strain* TOS07 (KP729643.2)
K.pneumoniae	1455	1447	98.8	99.1	*Klebsiella pneumoniae* strain X11 (MZ389264.1)
E.tarda	1486	1508	99.0	98.9	*Edwardsiella tarda strain* ETY (GQ180181.1)
B.cereus	1445	1430	98.3	97.7%	*Bacillus cereus* strain J55 (PV242267.1)
